# Symptom prevalence and burden, and the risk of depression among patients with advanced cancer attending two South African oncology units

**DOI:** 10.3332/ecancer.2022.1349

**Published:** 2022-01-27

**Authors:** Lindsay Farrant, Richard Harding, David Anderson, Linda Greeff, Reshma Kassanjee, R Krause, Zainab Mohamed, Jeannette Parkes, Liz Gwyther

**Affiliations:** 1 Division of Interdisciplinary Palliative Care and Medicine, Department Public Health and Family Medicine, Faculty of Health Science, University of Cape Town, Anzio Road, Observatory 7925, Cape Town, South Africa; 2 Cicely Saunders Institute of Palliative Care, Policy and Rehabilitation, King’s College London, Bessemer Road, London SE5 9PJ, UK; 3 Radiation Oncology, Dunedin Hospital, Southern DHB, 201 Great King Street, Dunedin 9016, New Zealand; 4 Social Worker in Private Practice, Andrag Street, Welgemoed 7530, Cape Town, South Africa; 5 Centre for Infectious Disease Epidemiology and Research (CIDER), Department Public Health and Family Medicine, University of Cape Town, Anzio Road, Observatory 7925, Cape Town, South Africa; 6 Division of Radiation Oncology, Department of Radiation Medicine, Faculty of Health Science, University of Cape Town, L-Block D-Floor, Room D-8, Groote Schuur Hospital, Observatory 7925, Cape Town, South Africa

**Keywords:** cancer, palliative care, symptoms, pain, depression

## Abstract

**Background:**

The incidence of cancer is predicted to increase globally by 47% between 2020 and 2040, largely in low and middle-income countries. The World Health Organisation and World Health Assembly recognise palliative care as an essential component of cancer care. The evidence of palliative care needs among South African oncology patients is sparse. This study aimed to describe the prevalence and burden of symptoms and the risk of depression amongst oncology patients with stage 3 or 4 cancer.

**Methods:**

Demographic and clinical data were collected and the Memorial Symptom Assessment Scale Short Form was used to measure the 7-day period prevalence of 28 physical and 4 psychological symptoms of patients receiving oncology care. The Centre for Epidemiological Studies Depression Scale was used to measure the risk of depression.

**Results:**

A total of *N* = 343 patients were recruited, of which *n* = 229 (66.8%) had stage 4 cancer. The mean number of symptoms was 11.56 (SD = 5.86). Pain and feeling drowsy/tired were the two most prevalent symptoms. *N* = 66 (19.3%) were at risk of mild depression and *n* = 27 (7.9%) for major depression.

**Discussion:**

Pain and depression persist in advanced cancer care despite the advances in policy and clinical education. Health services research must now focus on how to enact this in routine practice.

## Background

The International Agency for Research on Cancer forecasts a 47% increase in new cancer cases globally between 2020 and 2040 [1]. Globocan (2020) predicts an 89% increase in Africa for all cancer incidence by 2040 [2], with 1.34 million people dying of cancer in

Africa between 2020 and 2040 [3]. The World Health Organisation’s (WHO) 2018 mortality figures estimated 57,373 total cancer deaths in South Africa for that year [4]. During that year, 12,357 cancer patients were cared for by South African Hospices [5].

The projected increase in serious health-related suffering in the last year of life for people in low, lower-middle and upper-middle income countries will be largely driven by cancer (407%, 168% and 96%, respectively) [6, 7]. The WHO articulates palliative care as an essential approach to cancer care that focuses on enhancing the quality of life for patients and families [8]. In 2014, the World Health Assembly (WHA) passed resolution 67.19, recognising palliative care as an essential component of care for all patients with life-threatening disease [9]. Palliative care is defined by the WHO as: ‘*an approach that improves the quality of life of patients and their families facing the problems associated with life-threatening illness, through the prevention and relief of suffering by means of early identification and impeccable assessment and treatment of pain and other problems, physical, psychosocial and spiritual*’ [10]. Meta-analyses of the available evidence has shown palliative care to be highly effective in improving outcomes for advanced cancer patients [11–13].

The characteristics of suffering that are unique to patients in a particular region should inform care planning and delivery. There is, however, very little evidence for the unique characteristics of suffering amongst patients with advanced cancer in South Africa. The point prevalence of public hospital inpatients with active life-limiting disease in 2011 was 16.6%, 50.8% of who had a cancer diagnosis [14]. Patients with advanced cancer in sub-Saharan Africa receiving formal palliative care report higher symptom prevalence and burden than those in the developed world [15]. There are no studies to date that report the symptom prevalence among advanced cancer patients who are not accessing formal palliative care. An earlier published systematic review comparing data on the prevalence of symptoms between advanced progressive conditions found no data on cancer patients in Africa [16], and a systematic review of care for patients with advanced disease in Africa found almost no evidence on the need or effectiveness of care [17]. Evidence from Africa is essential, as palliative care needs may be context-specific [18]. The symptom profile (in terms of prevalence and burden) of South Africans with advanced cancer who have not accessed palliative care is unclear. The existing evidence from South African patients who receive formal palliative care suggests a high prevalence of physical and psychological problems [15], a need for better spiritual care [19] and a need for information about symptoms and their control [20, 21]. There is a body of evidence describing high prevalence of clinical depression in advanced disease [22] and the impact of depression on cancer progression in self-report physical outcomes, such as disability, pain and fatigue [23–25]. There is also a paucity of evidence for depression among cancer patients, although one South African study found that 36.6% of breast cancer patients (any stage) were at risk for depression [26]. The small body of existing evidence in Africa also includes cancer populations in West and East Africa [27–30]. In order to be able to provide ‘*feasible, acceptable and appropriate*’ [15, 17] palliative care to a growing number of patients, if the afore-mentioned predictions are accurate, we need data on the symptom prevalence and burden of patients with advanced cancer. No published study has aimed to determine the prevalence of depression, physical symptoms or quality of life in South Africans with advanced cancer.

Advances have been achieved in palliative care service provision in South Africa, which rely on a strong national network of accredited hospices, and the provision of medical and postgraduate education [31]. However, a systematic appraisal of the status of palliative care in sub-Saharan African countries found almost no evidence on the problems and outcomes of patients [17]. A study conducted at a hospital-based outreach palliative care service, in Soweto, found that hospital costs may be reduced by reducing the number of hospital admissions for those patients cared for by the palliative care service and that such services ‘*may improve quality of life*’ of the patients [32]. Our study aimed to measure the 7-day period prevalence and associated burden of physical and psychological symptoms among adults attending oncology clinics with stage 3 or 4 disease in the metropolitan Western Cape of South Africa, and to measure the risk of depression.

## Methodology

The primary sites were a public tertiary oncology unit and a private oncology unit. The study population included adult patients (aged 18 years or older) who had a diagnosis of cancer of stage 3 or 4 (or poor risk in the case of Kaposi sarcoma), attending both the inpatient and outpatient oncology services and who were able to consent to partake in the study. The eight primary cancer groups were selected from 2008 GLOBOCAN tables on age standardised incidence and mortality rates and local unpublished patient incidence data: breast, prostate, oesophageal, lung, cervical, colorectal, head and neck cancers and Kaposi’s sarcoma [33]. Recruitment tables of primary cancer were used in an effort to match samples between the two sites. We aimed for a total sample of 400 participants to measure the prevalence of any symptom with 95% confidence and 5% margin of error, proportionally stratified by primary malignancy using local (unpublished) data. Consecutive convenience sampling (December 2013 to March 2016) was applied. We excluded patients who were under 18 years old, unaware of their diagnosis, lacked capacity (either physical or mental) to give informed consent and complete a 45-minute interview.

As healthcare is becoming more responsive to patient accounts of their disease [34], patient reported outcome measures or ‘PROMS’ have become increasingly important in improving care and ensuring equity [35]. We selected measures with sound psychometric properties that have been previously used in South Africa. All tools, information and consent forms were forward and backward translated from English into the principle languages of isiXhosa and Afrikaans. All data were patient self-report. In order to reduce measurement and response bias, all questions were read aloud and patients gave their responses to be recorded by the trained researcher. The following patient demographics were collected: age, gender, public or private care, education and socio-economic status. The following disease-oriented variables were collected: primary cancer site, metastases, disease stage, human immunodeficiency virus status, current and previous treatment for the cancer and admissions in the prior 12 months. We also recorded place of care (inpatient or outpatient). The Eastern Co-operative Oncology Group Performance Scale (ECOG, 1 item) [36] was administered to measure physical performance. The Memorial Symptom Assessment Scale Short-form (MSAS-SF, 32 items) [37] was administered to measure the 7-day period prevalence and associated burden of multidimensional symptoms. The MSAS-SF offers three subscale indices of Physical Symptom Distress Index (MSAS-PHYS), Psychological Symptom Distress Index (MSAS-PSYCH) and Global Distress Index (MSAS-GDI). Each of these three subscales has a possible score range of 0–4. This well-validated multidimensional instrument captures the presence and distress of 28 physical and 4 psychological symptoms among cancer patients. The MSAS is one of the most widely used symptom tools in oncology, and we have previously applied it in multicentre research in South Africa [15]. The Centre for Epidemiological Studies on Depression (CES-D, 20 items) [38] was used as a screening scale measuring feelings and behaviours characteristic of symptoms of depression during the past week. The scores ranged from 0 to 60, and 16+ used as a cut-off for probable caseness [38]. It has been used in previous community depression studies in South Africa [39–41]. It has a Cronbach’s alpha value of 0.90 for women and 0.91 for men. None of the items in the selected measures mention palliative or terminal care, and therefore did not risk sharing information with patients that might not have been previously communicated by their clinician. The study was approved by the University of Cape Town’s Human Research Ethics Committee: HREC Ref No: 287/2012.

Sample characteristics were summarised descriptively. The CES-D scores were calculated and stratified according to the three-level cut-off, as proposed by Zich *et al* [42]. The MSAS-SF symptom prevalence and burden, as well as MSAS-SF subscales and total number of symptoms, were calculated. Analyses were conducted using Statistical Package for the Social Sciences Statistics (version 26).

## Results

### Sample characteristics

A total of *N* = 343 participants were recruited, with *n* = 48 (14%) in the private sector and *n* = 295 (86%) in the public sector. The majority were outpatients (*n* = 247, 72%). There were *n* = 201 (58.6%) female participants. The mean age of the participants was 58.4 years (SD 13.1). Ninety-three participants (27.1%) had completed primary school education, 205 (59.8%) had completed a secondary school education and 41 (12%) had a higher qualification. Ninety-five (27.7%) participants were receiving chemotherapy at the time of study and *n* = 56 (16.3%) were receiving radiotherapy, whilst* n* = 185 (53.9%) had previously received chemotherapy and *n* = 170 (49.6%) radiotherapy. The majority (*n* = 229, 66.8%) had been staged as 4. Table 1 details the primary malignancy diagnoses for participants. *N* = 20 participants were recruited with primary malignancies other than these 8 groups and were included in the overall analysis.

### Measures

The median score for the ECOG functional status was 2 (SD = 1.09). Forty-nine (14.3%) participants were fully functional (i.e., ECOG score = 0), 29.7% had an ECOG 1 score, 31.2% had an ECOG 2 score, 19.2% had an ECOG 3 score and 5.5% were completely disabled with an ECOG 4 score.

The 7-day period prevalence of symptoms and burden as per the MSAS-SF is detailed in Table 2. The mean number of symptoms was 11.56 (SD = 5.86). The ten most prevalent symptoms were pain (64.7%), feeling drowsy/tired (64.7%), lack of energy (62.4%), dry mouth (53.4%), worrying (51.3%), shortness of breath (50.1%), cough (48.1%), feeling sad (42.9%), changes in skin (42.9%) and difficulty sleeping (42.0%).

For participants reporting pain, 47.3% reported that their pain distressed them on the two worst response levels, i.e., ‘*quite a bit*’ or ‘*very much*’. Worry was the most prevalent psychological symptom, with 30.7% of the participants reporting worrying ‘*frequently*’ or ‘almost *constantly*’. Difficulty sleeping was reported by 144 (42.0%) participants and was the most burdensome symptom reported, with 61.7% of the participants with the symptom, reporting that this symptom distressed them ‘*quite a bit*’ or ‘*very much*’. Table 2 presents the 7-day period prevalence and burden of symptoms.

MSAS-SF subscales were as follows: MSAS-GDI was 1.23 (SD = 0.88), MSAS-PHYS was 1.09 (SD = 0.83), and MSAS-PSYCH was 0.91 (SD = 0.87).

According to the three-level cut-off of the CES-D (*n* = 342), *n* = 249 (72.8%) participants had no risk of depression, *n* = 66 (19.3%) participants were at risk for mild depression and *n* = 27 (7.9%) participants were at risk for major depression.

## Discussion

These data present symptom prevalence and burden and the risk of depression (measured by CES-D) amongst patients with advanced cancer who were receiving care in oncology clinics. Standard care in both private and public sectors included standard oncology care. Palliative care training had not yet been integrated into oncology registrar training and so although standard oncology care would have included some aspects of palliative care, this would not routinely have included integrated palliative care.

The mean number of symptoms experienced by patients in our study is similar to those of international studies, including studies in other African countries [43–48]. However, the study conducted by Harding *et al* [15] on patients with cancer receiving palliative care in Uganda and South Africa found a mean number of 18 symptoms and higher mean MSAS-SF subscale indices than in our study. It is possible that patients with more than the average number of symptoms, or with more advanced disease, are more readily referred to a palliative care service.

Studies report varying prevalence of pain, from 41% in a study of Chinese cancer patients [45] to 87% in a study of patients with cancer receiving palliative care in Uganda and South Africa [15, 43, 44, 47–49] with a systematic review reporting a prevalence of at least 50% in cancer patients. The prevalence of pain in our study was 64.7%, with 47.1% of patients with pain describing the burden as high (rated as ‘quite a bit’ or ‘very much’), emphasising the importance of regular pain assessment and management as part of oncology care patients with stage 3 and 4 cancer. Feeling tired/drowsy, lack of energy and dry mouth were highly prevalent symptoms with a burden of over 45% each, and are similar to the findings in other studies of advanced cancer patients [15, 44]. While it may be difficult to palliate these symptoms, their impact on quality of life should be explored holistically by the interdisciplinary team. Shortness of breath and cough were reported by around half the patients, similar to the study in Uganda and South Africa, [15] but the associated burden of 47.1% reporting that shortness of breath bothered them ‘quite a bit’ or ‘very much’ is higher than in the Uganda/South Africa study [15].

Psychological symptoms were prevalent amongst this group, with worrying as the most commonly reported psychological symptom (51.3%), and 30.7% of participants with this symptom reporting high distress (rated at ‘frequently’ or ‘almost constantly’). The prevalence of all four of the psychological symptoms in our study is similar to the findings by Hwang *et al* [44], however, is lower than reported in the study by Harding *et al* [15]. The symptom distress is similar for feeling sad and worrying; however, our study reports higher distress from feeling nervous or feeling irritable [15]. What appears to be clear is that psychological symptoms and distress are common in patients with stage 3 and 4 cancer and it is important to screen for this distress. There is some evidence that the prevalence of depression in the general population of South Africa is similar to, or perhaps somewhat higher than, that reported from developing countries [50–52]. Just over a quarter of our sample were at risk for depression, with 7.9% being at risk for major depression. This finding correlates with international literature on the risk for depression amongst patients with advanced cancer [44, 22]. Similarly, Kagee *et al* [26] reported one-third of South African breast cancer patients were at risk for depression, of which half had stage 2 disease. Pirl *et al* [53] found that treatment of depression in patients with recently diagnosed metastatic non-small cell lung cancer did improve their depression. This data regarding the high risk for depression amongst oncology patients in South Africa, together with evidence of the benefit of prompt treatment of depression, is a call to ensure adequate and regular screening and access to treatment and support for patients with advanced cancer who have depression or experience other psychological distress.

There are a number of limitations of this study. We were unable to reach the intended target sample size due to recruitment being limited to the funding available. We were unable to report a response rate because the study was introduced to participants by a variety of clinicians and the recording of accurate response rates in a busy clinic setting was challenging.

## Conclusion

These findings show that there is a high physical and psychological symptom burden and distress among advanced cancer patients and associated risk for depression. The international evidence for the benefit of the integration of a palliative care approach into oncology care for patients with advanced cancer is clear [11–13]. The findings verify the need to incorporate palliative care training into oncology programmes. The National Policy Framework and Strategy on Palliative Care 2017–2022 provides the platform for the necessary integration to occur [54], but resource allocation has been very limited. There is an urgent need to harness available resources for interdisciplinary teamwork between trained and equipped healthcare professionals, alongside the need for research into how to provide integrated ‘*feasible, acceptable and appropriate*’ palliative care [15, 17] for patients receiving care in oncology clinics. This includes the need for collaboration with mental health services for the holistic care of patients with depression. Our data demonstrate persisting high prevalence and burden of symptoms, despite enabling policy and clinical education. Mechanisms must be developed and evaluated to assist clinicians in incorporating palliative care into oncology practice.

## Authors’ contributions

LF wrote and all authors reviewed and commented on the manuscript. RH and LG conceived the study. RH, LG and LF wrote the study proposal with input from JP, DA, LGreeff. LF conducted statistical analysis with guidance from RK.

## Conflicts of interests

The authors declare that they have no competing interests.

## Funding

Funding for this study was from AORTIC and NIH in the form of a BIG CAT II award.

## Figures and Tables

**Table 1.** Primary malignancy (*n* = 343).

Primary cancer	Frequency	Percent (%)
Lung cancer	87	25.4
Breast cancer	87	25.4
Colorectal cancer	60	17.5
Head and neck tumours	29	8.5
Prostate cancer	23	6.7
Cervical cancer	15	4.4
Cancer of the oesophagus	12	3.5
Kaposi Sarcomas	10	2.9
Other	20	5.8

**Table 2.** The 7-day period prevalence of symptoms and burden by MSAS-SF.

7-day period prevalence of symptoms and burden (*n* = 343)						
		Burden of prevalent symptoms, *n* (%) ^a^				
Physical symptoms	Prevalence *N* (%)	Not at all (1)	A little bit (2)	Somewhat (3)	Quite a bit (4)	Very much (5)
Pain	222 (64.7%) (missing *n* = 1)	11 (5.0%)	67 (30.2%)	39 (17.6%)	38 (17.1%)	67 (30.2%)
Feeling drowsy/tired	222 (64.7%)	28 (12.6%)	54 (24.3%)	38 (17.1%)	37 (16.7%)	65 (29.3%)
Lack of energy	214 (62.4%)	21 (9.8%)	55 (2.6%)	41 (19.2%)	42 (19.6%)	55 (25.7%)
Dry mouth	183 (53.4%)	31 (16.9%)	41 (22.4%)	21 (11.5%)	37 (20.2%)	53 (28.9%)
Shortness of breath	172 (50.1%)	20 (11.6%)	47 (27.3%)	24 (13.9%)	29 (16.9%)	52 (30.2%)
Cough	165 (48.1%)	29 (17.6%)	48 (29.1%)	18 (10.9%)	24 (14.5%)	45 (27.3%)
Changes in skin	147 (42.9%)	56 (38.1%)	29 (19.7%)	14 (9.5%)	18 (12.2%)	30 (20.4%)
Difficulty sleeping	144 (42.0%)	16 (11.1%)	22 (15.3%)	17 (11.8%)	31 (21.5%)	58 (40.2%)
Constipation	141 (41.1%)	22 (15.6%)	32 (22.7%)	20 (14.2%)	26 (18.4%)	41 (29.1%)
Sweats	141 (41.1%)	33 (23.4%)	41 (29.1%)	12 (8.5%)	20 (14.2%)	35 (24.8%)
Numbness/tingling in hands or feet	136 (39.7%)	27 (19.9%)	32 (23.5%)	33 (24.3%)	18 (13.2%)	26 (19.1%)
Lack of appetite	136 (39.7%)	20 (14.7%)	28 (20.6%)	18 (13.2%)	25 (18.4%)	45 (33.1%)
Nausea	133 (38.8%)	15 (11.3%)	41 (30.8%)	24 (18.0%)	21 (15.8%)	32 (24.1%)
Changes in way food tastes	125 (36.4%)	29 (23.2%)	26 (20.8%)	19 (15.2%)	27 (21.6%)	24 (19.2%)
Weight loss	119 (34.7%)	41 (34.5%)	15 (12.6%)	3 (2.5%)	29 (24.4%)	32 (26.9%)
I don’t look like myself	119 (34.7%)	40 (33.6%)	25 (21.0%)	11 (9.2%)	22 (18.5%)	21 (17.6%)
Dizziness	119 (34.7%)	16 (13.4%)	33 (27.7%)	34 (28.6%)	10 (8.4%)	26 (21.8%)
Feeling bloated	93 (27.1%)	14 (15.1%)	21 (22.6%)	11 (11.8%)	24 (25.8%)	22 (23.7%)
Itching	84 (24.5%)	19 (22.6%)	23 (27.4%)	9 (10.7%)	8 (9.5%)	24 (28.6%)
Swelling of arms or legs	80 (23.3%)	26 (32.5%)	17 (21.3%)	8 (10.0%)	8 (10.0%)	23 (28.8%)
Difficulty concentrating	78 (22.7%)	12 (15.3%)	21 (26.9%)	22 (28.2%)	12 (15.3%)	12 (15.3%)
Difficulty swallowing	70 (20.4%)	1 (1.4%)	21 (30.0%)	9 (12.9%)	15 (21.4%)	24 (34.3%)
Vomiting	64 (18.7%)	12 (18.8%)	23 (35.9%)	4 (6.3%)	14 (21.9%)	11 (17.2%)
Diarrhoea	51 (14.9%)	10 (19.6%)	18 (35.3%)	8 (15.7%)	2 (3.9%)	13 (25.5%)
Problems with sexual interest/activity	50 (14.6%)	12 (24.0%)	8 (16.0%)	3 (6.0%)	3 (6.0%)	24 (48.0%)
Mouth sores	45 (13.1%)	7 (15.6%)	16 (35.6%)	9 (20.0%)	8 (17.8%)	5 (11.1%)
Hair loss	36 (10.5%)	19 (52.8%)	8 (22.2%)	1 (2.8%)	3 (8.3%)	7 (19.4%)
Problems urinating	35 (10.2%)	7 (20.0%)	7 (20.0%)	3 (8.6%)	6 (17.1%)	12 (34.3%)
**Psychological symptoms**	**Prevalence**	**Rarely (1)**	**Occasionally (2)**	**Frequently (3)**	**Almost constantly (4)**	
Worrying	176 (51.3%)	47 (26.7%)	75 (42.6%)	26 (14.8%)	28 (15.9%)	
Feeling sad	147 (42.9%)	57 (38.8%)	56 (38.1%)	20 (13.6%)	14 (9.5%)	
Feeling nervous	142 (41.4%)	42 (29.6%)	46 (32.4%)	34 (23.9%)	19 (13.4%)	
Feeling irritable	133 (38.8%)	35 (26.3%)	52 (39.1%)	27 (20.3%)	18 (13.5%)	

a Do not add up to 100% due to rounding.

## References

[1] Sung H, Ferlay J, Siegel RL (2021). Global cancer statistics 2020: GLOBOCAN estimates of incidence and mortality worldwide for 36 cancers in 185 countries. CA Cancer J Clin.

[2] Union for International Cancer Control (2020). GLOBOCAN: Cancer Tomorrow: Estimated Number of New Cases from 2020 to 2040.

[3] Union for International Cancer Control (2020). GLOBOCAN: Cancer Tomorrow: Estimated Number of Deaths from 2020 to 2040.

[4] World Health Organisation (2020). South Africa: Cancer Country Profile 2020.

[5] Hospice Palliative Care Association of South Africa (2020). HPCA Annual Report 2020.

[6] Sleeman KE, Brito M, Etkind S (2019). The escalating global burden of serious health-related suffering: projections to 2060 by world regions, age groups, and health conditions. Lancet Glob Health.

[7] Sleeman KE, Gomes B, de Brito M (2021). The burden of serious health-related suffering among cancer decedents: global projections study to 2060. Palliat Med.

[8] World Health Organisation (2021). Cancer: Palliative Care.

[9] Executive Board 134 Strengthening of Palliative Care as a Component of Integrated Treatment Within the Continuum of Care.

[10] Sepúlveda C, Marlin A, Yoshida T (2002). Palliative care: the World Health Organisation's perspective. JPSM.

[11] Gaertner J, Siemens W, Meerpohl JJ (2017). Effect of specialist palliative care services on quality of life in adults with advanced incurable illness in hospital, hospice, or community settings: systematic review and meta-analysis. BMJ.

[12] Higginson IJ, Evans CJ (2010). What is the evidence that palliative care teams improve outcomes for cancer patients and their families?. Cancer J.

[13] Seow H, Brazil K, Sussman J (2014). Impact of community based, specialist palliative care teams on hospitalisations and emergency department visits late in life and hospital deaths: a pooled analysis. BMJ.

[14] van Niekerk L, Raubenheimer PJ (2014). A point-prevalence survey of public hospital inpatients with palliative care needs in Cape Town, South Africa. S Afr Med J.

[15] Harding R, Selman L, Agupio G (2011). The prevalence and burden of symptoms amongst cancer patients attending palliative care in two African countries. Eur J Cancer.

[16] Solano JP, Gomes B, Higginson IJ (2006). A comparison of symptom prevalence in far advanced cancer, AIDS, heart disease, chronic obstructive pulmonary disease and renal disease. J Pain Symptom Manage.

[17] Harding R, Higginson IJ (2005). Palliative care in sub-Saharan Africa. Lancet.

[18] Afolabi OA, Nkhoma K, Maddocks M (2021). What constitutes a palliative care need in people with serious illnesses across Africa? A mixed-methods systematic review of the concept and evidence. Palliat Med.

[19] Selman LE, Higginson IJ, Agupio G (2011). Quality of life among patients receiving palliative care in South Africa and Uganda: a multi-centred study. Health Qual Life Outcomes.

[20] Murray SA, Grant E, Grant A (2003). Dying from cancer in developed and developing countries: lessons from two qualitative interview studies of patients and their carers. BMJ.

[21] Selman L, Higginson IJ, Agupio G (2009). Meeting information needs of patients with incurable progressive disease and their families in South Africa and Uganda: multicentre qualitative study. BMJ.

[22] Hotopf MCJ, Addington-Hall J, Ly KL (2002). Depression in advanced disease: a systematic review Part 1. Prevalence and case finding. Palliat Med.

[23] Hotopf MMR, Wadsworth M, Wessely S (1998). Temporal relationships between physical symptoms and psychiatric disorder. Results from a national birth cohort. Br J Psychiatry.

[24] Wells KB SA, Hays RD, Burman MA (1989). The functioning and well-being of depressed patients. Results from the medical outcomes study. JAMA.

[25] Barkwell D (1991). Ascribed meaning a critical factor in coping and pain attenuation in patients with cancer-related pain. J Palliat Care.

[26] Kagee A, Roomaney R, Knoll N (2018). Psychosocial predictors of distress and depression among South African breast cancer patients. Psychooncology.

[27] Boulaamane L, Essaadi I, Lalya I (2011). [Psychosocial impact of cancer on Moroccan adolescent and young adult: experience of National Institute of Oncology of Rabat]. Bull Cancer.

[28] Olagunju AT, Aina OF (2011). A controlled study of depression among attendees of an oncology clinic in West Africa. Int J Psychiatry Med.

[29] Clegg-Lamptey JNA, Dakubo JCB, Attobra YN (2009). Psychosocial aspects of breast cancer treatment in Accra, Ghana. East Afr Med J.

[30] Nuhu FT, Odejide O, Adebayo KO (2009). Psychological and physical effects of pain on cancer patients in Ibadan, Nigeria. Afr J Psychiatry.

[31] Ens C, Chochinov HM, Gwyther L (2011). Postgraduate palliative care education: Evaluation of a South African programme. S Afr Med J.

[32] Hongoro C, Dinat N (2011). A cost analysis of a hospital-based palliative care outreach program: implications for expanding public sector palliative care in South Africa. J Pain Symptom Manage.

[33] Union for International Cancer Control (2008). GLOBOCAN: Cancer Mortality Data South African Republic 2008.

[34] Giusti A, Nkhoma K, Petrus R (2020). The empirical evidence underpinning the concept and practice of person-centred care for serious illness: a systematic review. BMJ Glob Health.

[35] Dawson J, Doll H, Fitzpatrick R (2010). The routine use of patient reported outcome measures in healthcare settings. BMJ.

[36] Oken MM, Creech RH, Tormey DC (1982). Toxicity and response criteria of the Eastern Cooperative Oncology Group. Am J Clin Oncol.

[37] Chang VT, Hwang SS, Feuerman M (2000). The memorial symptom assessment scale short form (MSAS-SF). Cancer.

[38] Radloff LS (1977). The CES-D scale: A self-report depression scale for research in the general population. Appl Psychol Measure.

[39] Nduna M, Jewkes RK, Dunkle KL (2010). Associations between depressive symptoms, sexual behaviour and relationship characteristics: a prospective cohort study of young women and men in the Eastern Cape, South Africa. J Int AIDS Soc.

[40] Hutton HE, Lyketsos CG, Zenilman JM (2004). Depression and HIV risk behaviors among patients in a sexually transmitted disease clinic. Am J Psychiatry.

[41] Kaminer D, Grimsrud A, Myer L (2008). Risk for post-traumatic stress disorder associated with different forms of interpersonal violence in South Africa. Soc Sci Med.

[42] Zich JM, Attkisson CC, Greenfield TK (1990). Screening for depression in primary care clinics: the CES-D and the BDI. Int J Psychiatry Med.

[43] Tranmer JE, Heyland D, Dudgeon D (2003). Measuring the symptom experience of seriously Ill cancer and noncancer hospitalized patients near the end of life with the Memorial Symptom Assessment Scale. J Pain Symptom Manag.

[44] Hwang SS, Chang VT, Cogswell J (2004). Study of unmet needs in symptomatic veterans with advanced cancer: incidence, independent predictors and unmet needs outcome model. J Pain Symptom Manage.

[45] Lam WW, Law CC, Fu YT (2008). New insights in symptom assessment: the Chinese Versions of the Memorial Symptom Assessment Scale Short Form (MSAS-SF) and the Condensed MSAS (CMSAS). J Pain Symptom Manage.

[46] Bekelman DB, Rumsfeld JS, Havranek EP (2009). Symptom burden, depression, and spiritual well-being: a comparison of heart failure and advanced cancer patients. J Gen Intern Med.

[47] Lazenby M, Sebego M, Swart NC (2016). Symptom burden and functional dependencies among cancer patients in Botswana suggest a need for palliative care nursing. Cancer Nurs.

[48] Isiaka-Lawal SA (2017). An Exploration of Symptom Burden among Breast and Gynaecological Cancer Patients Accessing Care at University of Ilorin Teaching Hospital, Ilorin, Kwara State, Nigeria.

[49] Breivik H, Cherny N, Collett B (2009). Cancer-related pain: a pan-European survey of prevalence, treatment, and patient attitudes. Ann Oncol.

[50] Myer L, Stein DJ, Grimsrud A (2008). Social determinants of psychological distress in a nationally-representative sample of South African adults. Soc Sci Med.

[51] Tomlinson M, Swartz L, Kruger LM (2007). Manifestations of affective disturbance in sub-Saharan Africa: key themes. J Affect Disord.

[52] Hamad R, Fernald LC, Karlan DS (2008). Social and economic correlates of depressive symptoms and perceived stress in South African adults. J Epidemiol Community Health.

[53] Pirl WF, Greer JA, Traeger L (2012). Depression and survival in metastatic non-small-cell lung cancer: effects of early palliative care. J Clin Oncol.

[54] South African National Department of Health (2017). National Policy Framework and Strategy on Palliative Care 2017–2022.

